# Research on Damage Evolution Law of Glazed Hollow Beads-Cement/Sodium Silicate Grouting Materials under Different Cycles of Loading and Unloading

**DOI:** 10.3390/ma17010204

**Published:** 2023-12-30

**Authors:** Tao Liu, Weijing Yao, Jinxiu Han, Yu Liu, Heng Wang

**Affiliations:** 1 School of Civil Engineering and Architecture, Anhui University of Science and Technology, Huainan 232001, China; taoliu666@126.com (T.L.);; 2 Anhui Key Laboratory of Mining Construction Engineering, Anhui University of Science and Technology, Huainan 232001, China; 3 Postdoctoral Science Research Workstation, Wuhu Surveying and Mapping Design Institute Co., Ltd., Wuhu 241000, China

**Keywords:** grouting material, glazed hollow beads, sodium silicate, orthogonal test, cyclic loading and unloading

## Abstract

With the depletion of shallow resources, deep resource mining has become a trend. However, the high temperature and complex stress environment in deep mines make resource extraction extremely challenging. This paper developed a thermal insulation grouting material made of glazed hollow beads, sodium silicate, and cement and tested the compressive strength, gelation time, and stone rate under various curing days in light of the issue of high temperature heat damage in high ground temperature mines and the impact of mining on roadway grouting bolt support. Fatigue strength, fatigue deformation, load-residual strain, energy evolution and microscopic features were studied and analyzed in relation to the damage law of graded cyclic loading and unloading under the number of varying cycles. The findings demonstrate that cyclic loading and unloading strength is lower than uniaxial compressive strength. The fatigue strength is significantly decreased when the number of cycles reaches its limit. Residual strain is less sensitive to changes in stress than load strain. The fitting correlation coefficients of total output energy and elastic energy are higher than 0.71.

## 1. Introduction

High rock temperatures and thermal stresses present significant obstacles to the safe and effective extraction of deep resources as mining depths continue to rise [1]. For example, Carletonville Gold Mine in South Africa is known as the deepest metal mine in the world, with a depth exceeding 4000 m. The average depth of German coal mines in 2000 was over 1000 m, while the average depth of British coal mines in 2000 was 700 m, with the deepest reaching 1220 m. Heat damage prevention is an inevitable challenge in the extraction of deep resources because the mine’s high temperature environment endangers the health of the workers and lowers working efficiency and equipment lifespan [2]. The primary method of deep roadway support is grouting bolts [3]. However, because grouting materials are harmed by human excavation activities and are subject to cyclic load disturbance, there are significant hidden risks to the safety of construction workers. Research on mine heat damage treatment techniques and damage laws under cyclic load is therefore required [4,5].

Prior to taking any remedial action for mine heat damage, it is important to identify the cause of the damage [6]. Mine underground heat sources include surrounding rock heat release, mechanical and electrical equipment, compression heat release, transportation heat release, oxidative heat release, and other heat releases [7,8]. As mine depth increased, surrounding rock heat release accounted for a greater share of the source’s heat. In terms of mine heat damage control technology, there are currently two main facets [9,10,11]. The first is non-mechanical heat damage control technology, which primarily entails ventilation cooling, heat insulation, and personal protection, among other things. The widespread use of variable speed drive (VSD) technology presents a significant opportunity to improve energy efficiency for ventilation cooling. After performing an energy assessment on 20 large mine cooling systems, Gideon Edgar Du Plessis evaluated potential savings and feasibility indicators [12]. A pilot implementation study was conducted on a mine as well to empirically verify the anticipated savings. The results of the pilot trial, the projected cost reductions, and the audit findings are all provided. It has been proven that it is economically viable to install VSDs in the pumps and fans of mine cooling systems on a large scale. A total of 144,721 MW h, or 32.2%, of the annual electrical energy consumption can be saved. Han Qiaoyun evaluated the thermal comfort of the cooling system [13]. Using on-site experimentation, numerical modeling, and the WBGT (wet bulb globe temperature index) index, the cooling effect of the HEMS (high temperature exchange machinary system) system was measured. The results indicated that all of the tunnel workers’ needs would not be met by the air cooler’s current outlet parameters, which are outlet air temperature, relative humidity (RH), and velocity, which are 18 °C, 80%, and 1.5 m/s, respectively. RH levels of 75% in the inlet tunnel and 60% in the mining tunnel, on the other hand, would be adequate. The results would assist in optimizing cooling in deep coal mines. The most direct method of heat insulation and grooming is to isolate the heat source, and the main goal of the heat source isolation method is to cover the surface of the nearby rock to stop the heat from the nearby rock from spreading to the inside of the roadway, which is an important auxiliary measure to improve the mine’s cooling efficiency and lower the cooling energy consumption of the equipment. His work focuses on the creation of materials for thermal insulation. Installing thermal insulation gunite (TIG) in the roads is an effective way to reduce local thermal hazards in mines. The development of mining TIG materials is the foundation of thermal insulation technology. The relatively high in situ stress, high geo-temperature, and relative humidity in deep mines, however, preclude the use of several modern and conventional insulating materials. A type of fly ash-inorganic mineral TIG material developed by Junhui Wang was used to simulate a high geo-temperature road [14]. Both the stability of the temperature field surrounding the nearby rocks and the wall’s capacity to dissipate heat are significantly impacted by the TIG layer. Additionally, the thermal flux densities tend to be constant once the TIG roadway starts to be ventilated, indicating that temperature disturbance has stopped. The other is mechanical heat damage control technology, which uses machine ice, cold air, and cold water to send them through a pipeline to each cold face. This method has a good cooling effect but is best suited for deeper mines due to its expensive equipment purchase, maintenance, and use costs. There are also some new cooling technologies, such as heat, electricity, cooling trigeneration technology, whose main goal is to extract gas from mines, use gas combustion to produce electricity, cool gas generator sets, and cool exhaust gas waste heat using lithium bromide absorption refrigeration units, while using gas power generation to use the remaining heat to prevent mine heat damage. The HEMS cooling system technology uses the rushing water to extract the cooling amount, which is then directly exchanged with the hot air on the working surface to produce the cooling effect [15]. Geothermal energy is a green, low-carbon, recyclable renewable energy source with significant potential for energy supply, energy conservation, and emission reduction. In a similar vein, mine production uses geothermal energy as the target of control and prevention. Its use can be broken down into two categories: direct use and power production [16]. Direct use includes things like heating, cooling, healthcare, hot spring bathing, tourism, aquaculture, greenhouse gardening, et al.

The impact of cyclic loading and unloading on the mechanical characteristics of materials has been extensively researched. In order to cause the sandstone specimens to have varying degrees of damage, Shoudong Xie used various upper stress limits to perform cyclic loading and unloading on them [17]. Then, he studied the mechanical behavior of damaged sandstone under various stresses. The strength change and energy development law of sandstone with different damage degrees were studied. Next, the damage of the loaded sandstone was quantitatively described. In order to comprehend how cyclic mining stress affects coal seams that contain gas, Changbao Jiang carried out an experimental investigation on the seepage characteristics, acoustic emission (AE) characteristics, and energy dissipation of coal under tier-loaded cyclic loading [18]. After the initial 10 MPa loading, the loading increases by 2.5 MPa for each stage. The terms permeability recovery rate, damping ratio, AE energy rate, and ring count rate were used to describe this process. A new equation was created for the damage variable of coal with dissipation energy.

At present, the main thermal insulation method is to cover a layer of thermal insulation material on the surface of rock [19]. Due to the limitation of roadway space, the thickness of thermal insulation layer is often small, so it has poor performance in thermal insulation and support effect. For cyclic loading and unloading tests, most scholars use fixed load cycles and graded loading cycles, and few scholars consider the effect of changing the number of loadings per stage on the mechanical properties of materials. In view of the shortcomings of the above research, a new type of thermal insulation grouting material was developed by mixing glazed hollow beads, sodium silicate and cement. The thermal insulation layer with large thickness was established, and the mechanical law of cyclic loading and unloading of the new material under different loading times was studied.

## 2. Test Materials and Methods

### 2.1. Raw Materials

**Figure 1:**
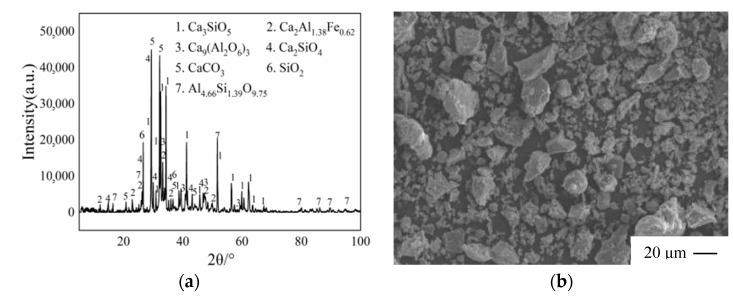
Physical properties of cement:(**a**) XRD analysis; (**b**) SEM analysis of cement.

**Figure 2:**
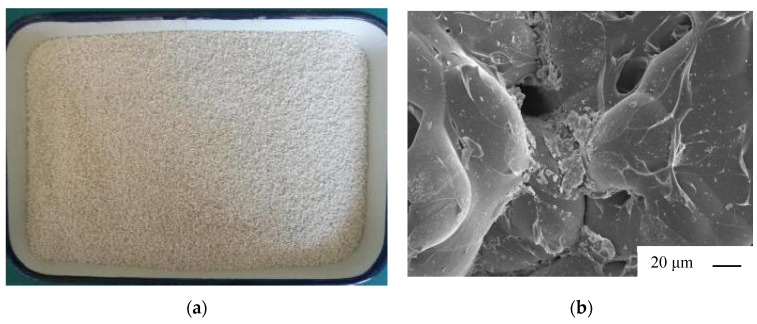
Pictures of glazed hollow beads: (**a**) real picture; (**b**) microscopic picture.

(1) The cement utilized in this test is PC 42.5 composite Huainan Bagongshan cement from Huainan building material Limited Company, Huainan, Anhui Province, China, with compressive strengths of 29.99 MPa and 49.75 MPa after 3 and 28 days, respectively. Table 1 displays the technical parameters, whereas Figure 1 displays X-ray Diffraction (XRD) and SEM examination.

(2) The physical and mechanical properties of the glazed hollow beads manufactured by Henan Jinhualan Mining Limited Company, Jinhua, Hunan Province, China, are listed in Table 2, and their actual image is depicted in Figure 2. It is composed of mineral grains that have been carefully selected and heated in an electric boiler. Under the puffing mode, the surface of the product is melted, the apertures are sealed, and irregular spherical particles are created by managing temperature and raw material empty time with precision. Due to the internal porous cavity structure, vitrified and closed surface, smooth gloss, and vitrification, the physical and chemical properties are extremely stable. The density of the glazed hollow beads chosen for this paper is 100 kg/m^3^.

(3) The Jiashan County Yourui Refractory Material Limited Company in Jiaxin, Zhejiang Province, China, produces sodium silicate. In the high-pressure steamer, the pressure steaming reaction is conducted under two to three atmospheres to produce liquid sodium silicate directly from quartzite powder and caustic soda. Table 3 contains a listing of the physical characteristics. According to this study, the density of sodium silicate is 1480 kg/m^3^.

(4) Water is general tap water.

### 2.2. Experimental Design

In order to obtain the most suitable ratio of new thermal insulation grouting materials, an orthogonal test was designed, and three influencing factors were chosen: the content of glazed hollow beads (factor B), the volume ratio of sodium silicate to cement slurry (factor C), and the ratio of water to cement (factor A). Each of these factors was divided into four levels, and the orthogonal table was L_16_ (4^5^). The specific factors and levels are listed in Table 4.

**Figure 3:**
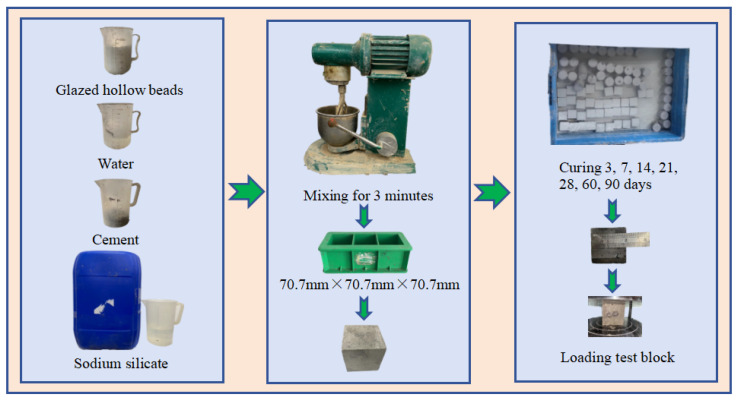
Flow chart of injection and test.

The test process is shown in Figure 3. The test block is divided into seven groups, which are used to test and cure 3, 7, 14, 21, 28, 60, 90 days after glazed hollow beads-cement/water glass thermal insulation grouting compressive strength [20,21], each large group is divided into 16 groups, each group has three test blocks, a total of 336 pieces, compressive strength specimen size of 70.7 mm × 70.7 mm × 70.7 mm. When preparing the specimen, the glazed hollow beads are first pre-wetted, the soaking time is 1 h, the prepared slurry is repeatedly poured back in two beakers by the cup pouring method so that the slurry no longer flows in the gelation time, the mould is removed 24 h after the specimen is made, and cured in standard supersaturated Ga(OH)_2_ solution at room temperature of 20 ± 2 °C, and the uniaxial compressive strength of the specimen is tested, and the ratio of the initial volume of the test block to the volume of the test block after 28 days of curing was used as the stone rate.

**Figure 4:**
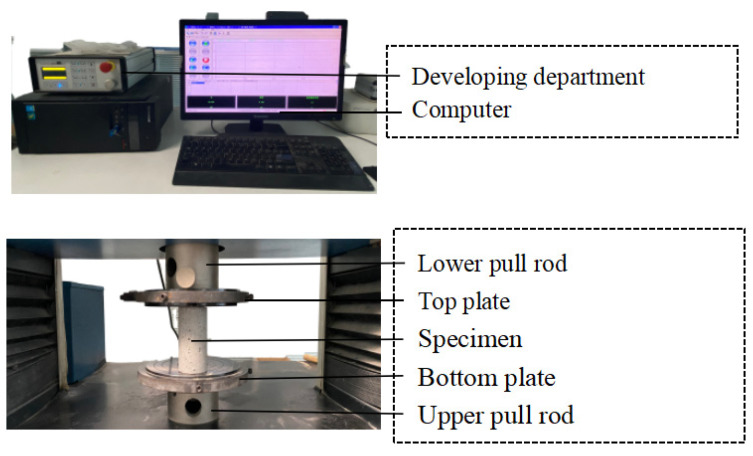
The RDL-200 rock compression rheometer.

To comprehensively analyze the fatigue characteristics evolution of grouting materials in complex surrounding rock environments [22]. The grading and unloading test of variable loading times is carried out on the specimens of the best ratio of heat insulation grouting materials. Figure 4 shows the RDL-200 rock compression rheometer used in the test. For cyclic loading and unloading of cylinders with specimen sizes of Φ 50 mm × 100 mm, in the experiment, six different loading paths were designed, namely the uniaxial compression path and cyclic loading path, in which the number of cyclic loading times was divided into 1, 2, 4, 6, and 8 cycles. In uniaxial compression tests, a rate of 1 mm/min is used to load the test piece to failure. In the cyclic load-unload test, stress control was used. The loading rate and unloading rate were 0.2 MPa/s, and the lower limit of unloading stress was always maintained during the load-unloading process, and both were discharged to 0 MPa. The upper limit of the loading stress is different [23]. The first stage of cyclic loading stress is 2.17 MPa, which is about 50% of the monotonic loading strength, and the amplitude of each subsequent stress increase is set to 0.43 MPa until the sample damage test stops.

## 3. Results and Analysis

### 3.1. Orthogonal Test Results

The compressive strength of each group of test blocks, gelation time and curing under standard conditions for 3 days, 7 days, 14 days, 21 days, 28 days, 60 days and 90 days were tested. The test results of glazed hollow beads-cement/water glass thermal insulation grouting under different curing times are shown in Table 5.

According to an indoor test, the optimal ratio of the new thermal insulation grouting material was determined. The ratio of water-cement was 0.6, the density of glazed hollow beads was 45 kg/m^3^, and the volume ratio of sodium silicate and cement slurry was 0.6. The thermal conductivity of the optimal group and cement slurry was measured at 0.93 and 0.41 W/(m·K), respectively. From the experimental results, it can be concluded that the new thermal insulation grouting material has better thermal insulation effect, higher stone rate and shorter gelation time.

### 3.2. Fatigue Strength

The peak stress in the uniaxial compression test is recorded as the uniaxial compressive strength (*f_cc_*), the corresponding strain is denoted as the peak strain (*εf_cc_*), and the pressure at failure in the cyclic load-unload test is designated as the fatigue failure stress (*f_p_*), the corresponding strain is the fatigue failure strain (*εf_p_*), according to the test results for the six loading paths. The fatigue coefficient (*K_p_*) is defined as the ratio of the uniaxial compressive strength to the fatigue failure stress of specimens with various loading times, as shown in Equation (1).

(1) $$K_{p}=\frac{f_{p}}{f_{cc}}$$

**Figure 5:**
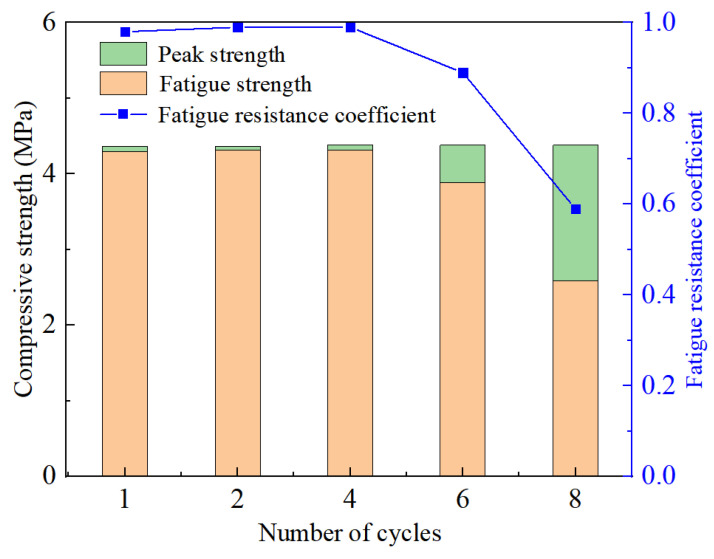
Fatigue failure stress of grouting material specimens with different number of loading cycles.

Figure 5 depicts the variation law of the fatigue failure stress and uniaxial compressive strength of grouting material specimens under various loading conditions. The grouting material in the loading-unloading process causes fatigue failure, resulting in cyclic loading and unloading strength being lower than uniaxial compressive strength. The increase in loading-unloading times also affects fatigue strength, as can be seen from Figure 5. The compressive strength is decreased by 2%, 1%, 1%, 11%and 41%, respectively, when the number of loading cycles is 1, 2, 4, 6, and 8. When the number of loading cycles does not reach the critical value, increasing the number of cycles has a minor impact on fatigue failure strength [24]. However, when the number of loading cycles reaches the critical value, the fatigue strength will be drastically reduced. Therefore, it is essential to research the number of loading cycles.

### 3.3. Fatigue Deformation

The grouting material’s soil specimen exhibits an unavoidable plastic deformation when subjected to cyclic loading and unloading, which can be attributed to the fact that it is not inherently an ideal elastic material [25]. As a result, in the process of compression failure, its deformation consists of irreversible plastic deformation and reversible elastic deformation, the latter of which can be recovered after unloading. The stress-strain curve reflects this characteristic, showing that a hysteresis loop cannot form when the loading and unloading curves do not coincide.

**Figure 6:**
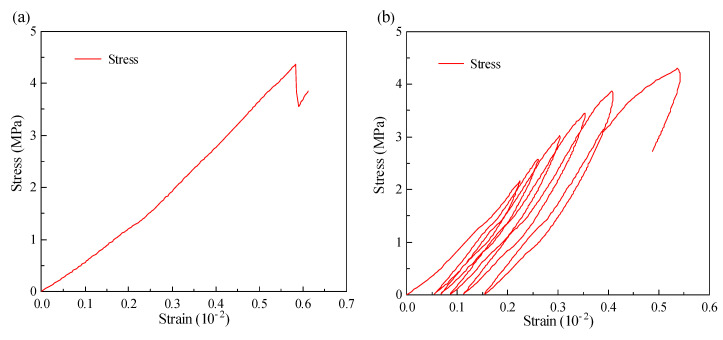

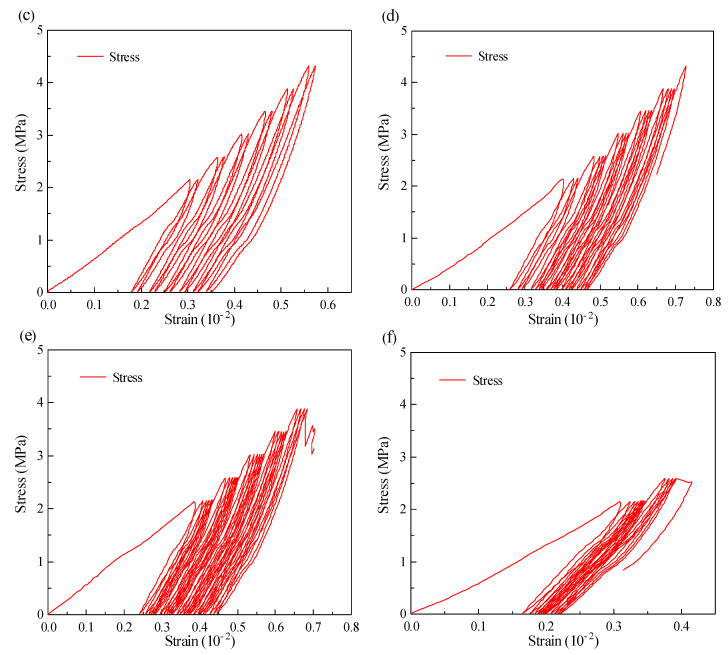
Different the number of loading cycles and load stress-strain curves for standard 90-day curing grout materials: (**a**) Uniaxial compressive strength; (**b**) 1 cycle; (**c**) 2 cycles; (**d**) 4 cycles; (**e**) 6 cycles and (**f**) 8 cycles.

Under uniaxial compression and cyclic loading without exceeding the number of loading cycles, the stress-strain curve is shown in Figure 6. As the amount of loading stress rises for a single cycle, the hysteresis area and width of the process become increasingly important. Excluding the last and pressure groups, for multiple cycles, the curve is initially sparse and then dense under each cycle load, and the deformation of the specimen primarily happens in the first cycle, which is depicted as a large opening on the curve [26]. This is because each increment of cyclic stress will generate fatigue cracks and significant deformation, and with subsequent stress cycling, only the gaps and cracks will be compressed, resulting in relatively small specimen deformation. This leads to a tendency for the hysteresis curve density to stabilize. The hysteresis curve becomes sparser and thinner as cyclic loading stresses get closer to failure, such as during the final few cycles. When the number of loading cycles reaches a specific value, the fatigue compressive strength decreases rapidly because the fatigue failure of the specimen occurs under multiple loading.

Figure 6’s first and last stress curves show that the initial loading curve is concave in the last stage stress, meaning that even as the strain gradually increases, the stress is always kept at a low level, which corresponds to the process of compaction and sealing of internal pores, which is referred to as the compaction stage [27]. The specimen is moving toward a dense state as a result of the gradual compaction of the pores, and the stress-strain curve growth exhibits a linear change trend, indicating that it is in an elastic stage. The strain growth rate is then noticeably accelerated as a result of the ongoing stress increase, and the curve top takes on the shape of a spindle. This shows that the loading process at this point has caused the internal pores to rupture along with the development of microcracks, which is known as the crack development stage. The grouting material specimen only goes through the compaction and elastic stages under higher stress, however, because of the low upper loading limit level, and the top of the curve is sharply angled.

**Figure 7:**
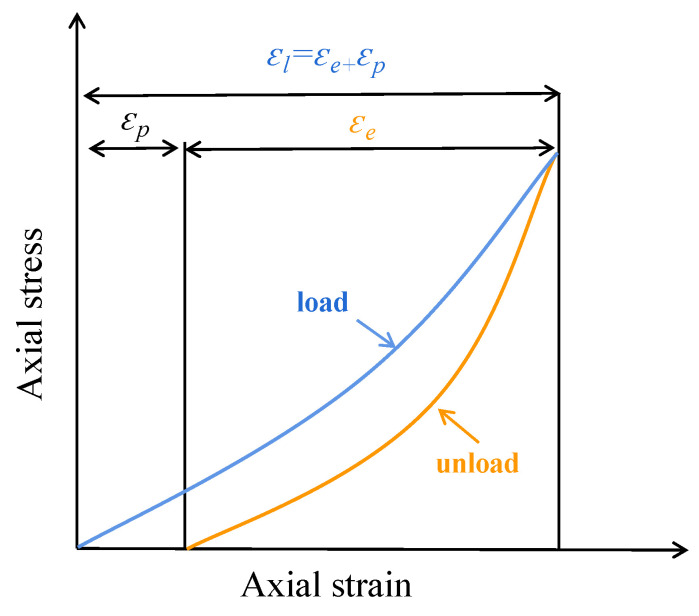
Schematic diagram of the strain relationship.

In fatigue experiments, axial strain is a very important damage indicator. In this test, the axial strain generated during a single cyclic loading is called the loading strain (*ε_l_*), and the axial strain generated during unloading is called the residual strain (*ε_p_*), and the difference between the two is the elastic strain (*ε_e_*) during the loading-unloading process, and the relationship between the loading strain, elastic strain and plastic strain is shown in Figure 7 and can also be expressed by Equation (2).

(2) $$\varepsilon_{l}=\varepsilon_{e}+\varepsilon_{p}$$

**Figure 8:**
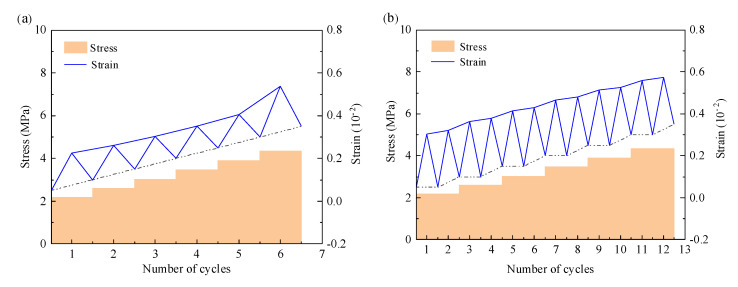

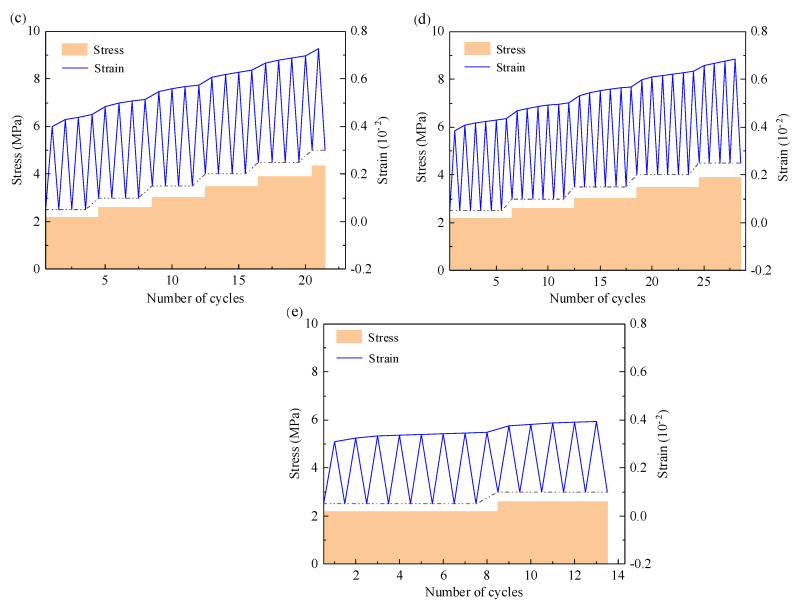
The relationship between stress-strain and the number of loading cycles of each group of specimens: (**a**) 1 cycle; (**b**) 2 cycles; (**c**) 4 cycles; (**d**) 6 cycles and (**e**) 8 cycles.

The change in axial strain with the number of loading cycles of a standard 90-day curing grouting material specimen is depicted in Figure 8, where blue represents the strain and cyclic loading process and yellow represents the stress loading process [28]. The figure shows that in the single cycle loading-unloading process, the early stress and strain are linearly related, but when the specimen is close to failure, the linear relationship breaks and the strain increases rapidly until failure. Because the specimen is more solid in structure after multiple loading, the strain increases with the cycle in the same stage of stress level, and the strain first increases and then decreases.

### 3.4. Loading-Residual Strain

**Figure 9:**
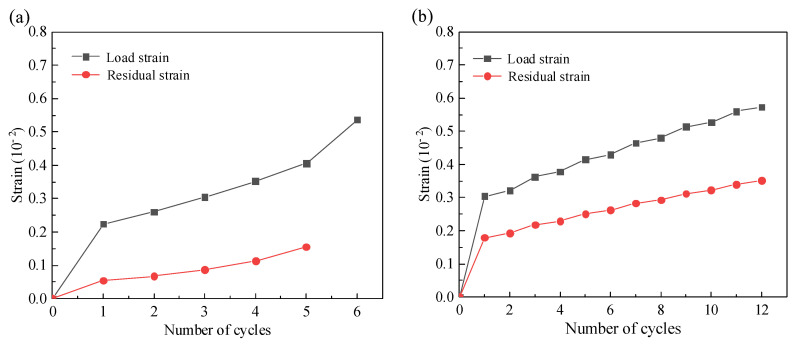

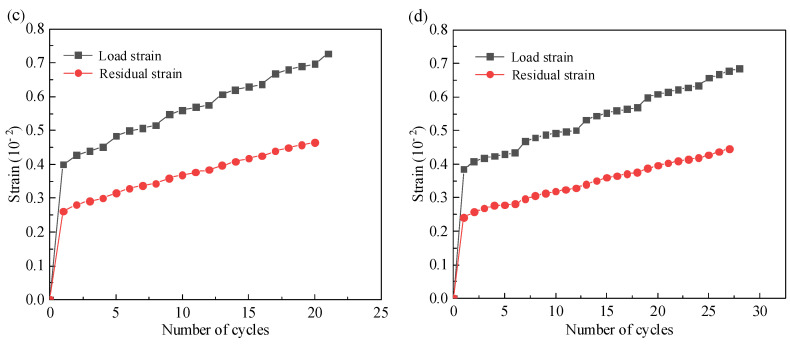
The load strain and residual strain of each group of specimens: (**a**) 1 cycle; (**b**) 2 cycles; (**c**) 4 cycles and (**d**) 6 cycles.

The loading strain and residual strain curves of the grouting material are shown in Figure 9, which grow after repeated loading-unloading and create a dramatic increase as the stress level increases [29]. In addition to the last stage of cyclic stress, when in the same cyclic stress level, the first cyclic load strain increases the most, and then gradually slows down to stability, in the final breaking stage, the strain increases rapidly, the specimen produces fatigue failure, and the specimens with the number of load cycles of 1, 2, 4, 6 and 8 of each stage are cycled 6 cycles in the cycle to the sixth stage, 12 cycles to the sixth stage, and 21 cycles to the sixth stage, destruction occurs when there are 24 cycles to the fifth stage and 14 cycles to the second stage.

Changes in residual strain can be indicative of irreversible deformation accumulation during cyclic loading and unloading. The residual strain continues to increase during repeated loading-unloading for different times of cyclic loading, with the greatest increase at the first load, as the first loading at each stage creates new cracks and voids, resulting in increased strain. At the same time, because the internal structure of the specimen is more solid after multiple peer loading, the rate of residual strain increase gradually decreases.

When the loading strain and residual strain are compared, it is discovered that the loading strain is more sensitive to stress changes than the residual strain, and the relationship between residual strain and stress is more linear, whereas the loading stress exhibits a stepped increase, indicating that when the stress is abrupt, the residual strain accounts for a smaller part of the loading strain, and the elastic strain accounts for the majority [30].

### 3.5. Energy Evolution

During the cyclic load-unloading, the grouting material specimen always exchanges energy with the surrounding system. This energy is typically continuously input from the outside into the grouting material in the form of mechanical energy, a portion of which is accumulated inside the specimen as elastic energy, while the remainder is consumed to cause irreversible plastic deformation [31]. Energy dissipation effectively reflects the entire process of internal defect compacting and new cracks developing and expanding until the specimen loses its strength. As a result, studying the failure process of grouting materials from an energy standpoint can better reflect the critical properties of their failure.

#### 3.5.1. Energy Density

In this paper, the cyclic loading-unloading process of grouting materials is investigated using energy density to eliminate the effect of specimen volume and size on axial strain. Assuming no heat exchange occurs between the specimen and the outside world during the test, the total energy density produced by the external force can be expressed as follows using the laws of conservation of energy and thermodynamics:

(3) $$U=U_{E}+U_{D}$$

where *U* denotes the total energy density, i.e., the energy density absorbed by the specimen. The elastic energy density stored inside the specimen is represented by *U_E_*. *U_D_* denotes the dissipative energy density, which includes friction between particles within the specimen as well as the energy density consumed by crack formation.

**Figure 10:**
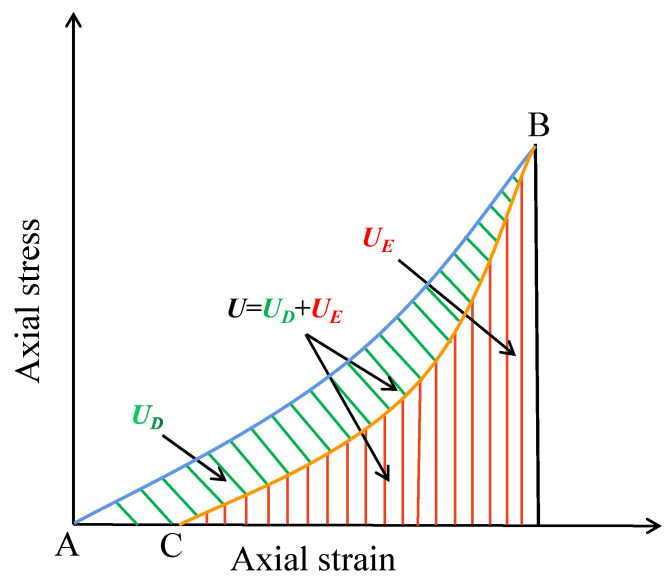
Calculation schematic diagram of cyclic loading and unloading energy density.

Figure 10 depicts a diagram of the energy density calculation. Point A is the starting point of loading, point B is the maximum point of loading, and point C is the lowest point of unloading. The total energy density *U* is denoted by the area around the loading curve AB and the abscissa, and the elastic energy density *U_E_* is denoted by the area around the unloading curve BC and the abscissa. The difference between the total energy density and the elastic energy density, that is, the area around the curve ABC and the abscissa, is the dissipative energy density *U_D_*. The energy dissipation process, according to the second law of thermodynamics, is unidirectional and irreversible, resulting in the propagation of cracks within the specimen and an increase in plastic deformation. The elastic energy accumulated in the specimen, on the other hand, is reversible, and as the elastic deformation increases during loading and unloading, a large amount of elastic energy is accumulated in the specimen, and this elastic energy is released during unloading [32]. The calculation formula of dissipation energy is as follows:

(4) $$U=\int_{\varepsilon_{A}}^{\varepsilon_{B}}\sigma_{U}d\varepsilon$$

(5) $$U_{E}=\int_{\varepsilon_{B}}^{\varepsilon_{C}}\sigma_{E}d\varepsilon$$

(6) $$U_{D}=U-U_{E}$$

where *σ_U_* is the load strain; *σ_E_* is the unload strain; *U* is the total energy density, MJ·m^−3^; *U_E_* is elastic energy density, MJ·m^−3^; *U_D_* is the dissipated energy density, MJ·m^−3^.

In addition, in the stages of cyclic loading-unloading, the energy dissipation of the grouting material specimen is mainly used for the development of irreversible plastic deformation in order to further illustrate the development of internal defects of the grouting material and the propagation process of cracks, this paper defines the ratio of dissipated energy density to total input energy density under each cycle as *η* energy dissipation ratio, the specific calculation formula is Equation (7), and the energy dissipation ratio *η* reflects the ability to dissipate energy in the concrete specimen [33]. A larger coefficient indicates that more energy is consumed. Otherwise, less energy is consumed. In addition, this coefficient can be used to characterize the strength of energy dissipation.

(7) $$\eta =\frac{U_{D}}{U}$$

**Figure 11:**
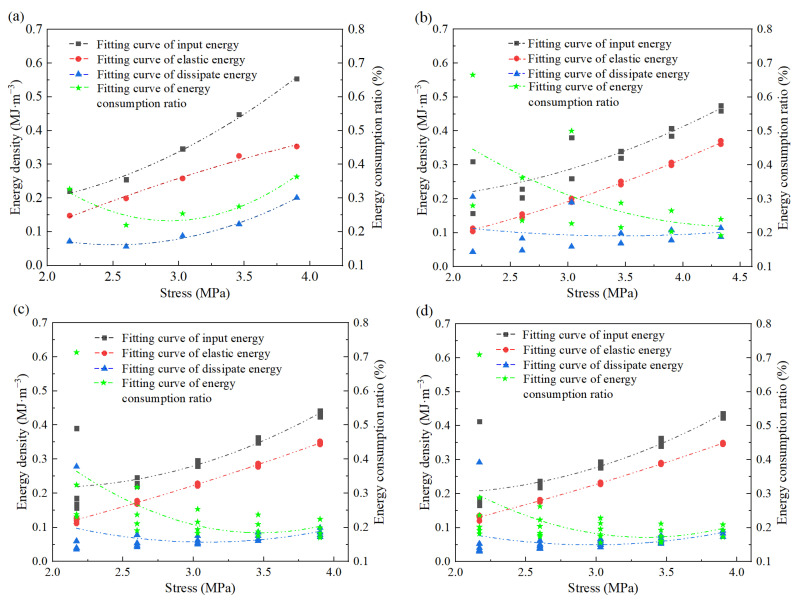
Energy evolution of grouting material specimens with different loading cycles: (**a**) 1 cycle; (**b**) 2 cycles; (**c**) 4 cycles and (**d**) 6 cycles.

The total energy density, elastic energy density, and dissipated energy density are often respectively referred to as input energy, elastic energy, and dissipated energy. Figure 11 shows the energy evolution law of grouting materials with different loading cycles.

Figure 11 shows that the various energy densities and energy dissipation ratios of different cycles are consistent, the input energy density and elastic energy density increase at different rates with the increase of stress level, and the dissipative energy density and energy dissipation ratio decrease first and then increase with the increase of stress level.

The energy change law of the grouting material can be loosely separated into three stages based on the change curve of each energy density parameter. initial pore compaction stage I, elastic deformation stage II, and plastic deformation stage III [34].

I stage: During the initial phase of stress loading, each energy parameter’s change curve varies gradually, and this stage mostly refers to the compaction of pores and cracks. This is because the specimen has several initial pores and fractures, and the maximum first-stage cyclic stress is around 50% of the maximum uniaxial compressive strength, at which point all of the specimen’s primary cracks tend to close and result in significant deformation. Only a small portion of the total energy is transformed into elastic potential energy and accumulated in the specimen, and the energy consumption ratio is high because the majority of the total energy is dissipated as irreversible deformation of voids and fractures. During the process of stress increase, the original crack has a tendency to contract and close, the specimen has a tendency to compact, and the amount of energy dissipated decreases as stress increases.

II stage: The loading stress increases during the cyclic loading-unloading process, and the grouting material enters the elastic deformation stage [35]. During this stage, the total input energy, elastic energy, and dissipative energy increase linearly, while the proportion of dissipated energy remains essentially unchanged, and the external input energy is primarily used for the specimen’s elastic deformation.

III stage: The grouting material specimen starts to exhibit plastic deformation as the stress gradually rises to the peak load. The early cycle loading and unloading damaged the specimen due to fatigue, which led to a quick increase in total input and a quick development of specimen deformation. The input energy is quickly transformed into dissipative energy as a result of the rapid growth and expansion of new cracks, and the amount of dissipated energy gradually rises.

The fitting results of the relationship between energy parameters and loading stress of different cycles are listed in Table 6, and it can be seen from the table that the total output energy and elastic energy fitting correlation coefficients are both high, both above 0.71, but the dissipative energy and energy consumption ratio fitting correlation coefficient is low, indicating that the total input energy density and elastic energy density basically increase [36]. Total input energy density increases the fastest with increasing stress level, followed by elastic energy density, dissipated energy density, and energy dissipation ratio under the same stress load. The value of each cycle fluctuates significantly in the early stages but tends to stabilize in the middle and late stages. The dissipative energy density and energy dissipation ratio decrease first and then increase with increasing stress. The deformativity density increases first and then decreases with increasing stress.

#### 3.5.2. Energy Dissipation

(1) Energy Consumption Ratio

**Figure 12:**
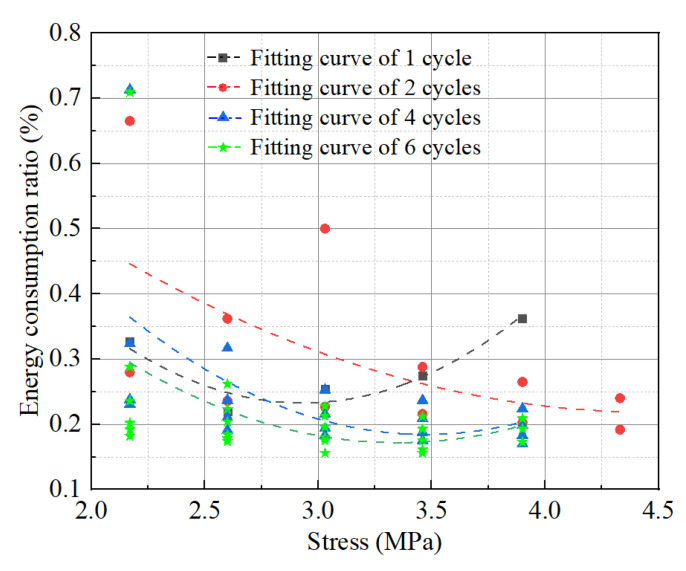
Energy consumption ratio of test pieces with different loading cycles.

The energy consumption ratio of the grouting material for various loading cycles is shown in Figure 12.

The energy consumption ratio curve of the grouting material specimen under various cycles can be seen in Figure 12, and it essentially exhibits a “U” shaped change trend [37]. Except for the load cycle of each stage, where the fluctuation range of the energy consumption ratio is smaller the more times the grouting material is loaded, indicating that after repeated loading-unloading, the interior of the specimen is gradually compacted and the average dissipation energy is reduced. Under the same stress, the energy consumption ratio fluctuation range of each cycle is large in the first few stages and relatively small in the later stages.

The cyclic stress at the end of stage I for grouting material specimens with 1, 4, and 6 loading times is approximately 3.03 MPa, which is roughly 70–80% of the uniaxial compressive strength of the grouting material specimen. Before the cyclic stress reaches about 3.03 MPa, the proportion of dissipated energy gradually declines while the proportion of elastic energy gradually increases [38]. The dissipated energy ratio starts to rise at about 3.03 MPa under cyclic stress over 3.03 MPa as a result of the loading process at higher stress levels causing the development of a large number of new cracks, which spread out and staggered with the original cracks, leading to a significant increase in plastic deformation.

(2) Energy Competition Coefficient

**Figure 13:**
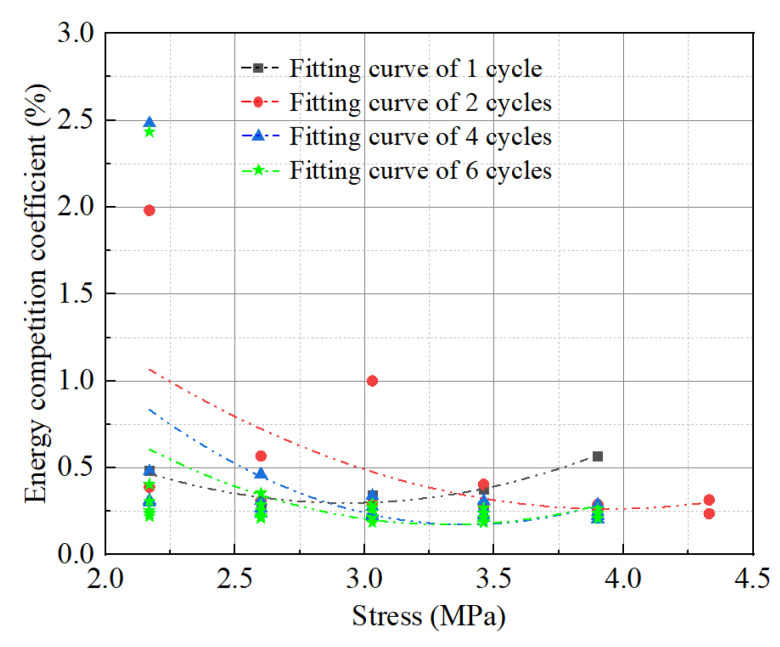
Energy competition coefficient.

The ratio of the dissipated energy density to the elastic energy density of each cyclic loading and unloading specimen is defined as the energy competition coefficient to characterize the energy competition relationship between the two. Figure 13 is the energy competition coefficient of different cyclic loading and unloading specimens.

As seen in Figure 13, loading cycles 1, 2, 4, and 6 demonstrate that, prior to stages 1, 3, and 2 of stress, respectively, the dissipative energy density increases with stress at a rate slower than the elastic energy density does.

The difference between the maximum competition coefficient and the minimum competition coefficient of all groups of specimens at the same stage is the total difference, and the difference between the maximum competition coefficient and the minimum competition coefficient of the individual group is the stage difference. As shown in Table 7, a single load of specimens with different loading cycles is significantly lower than multiple loading, possibly due to a single load’s limited fluctuation range. For multiple loading, 92% of the total difference in the competition coefficient comes from the difference of the first stage, and it can be seen that the first stage is the abrupt stage of the ratio of dissipative energy density and elastic energy density, which is primarily due to the specimen being compacted at this stage, the internal voids and cracks are reduced, and the plastic deformation is large, resulting in a slowdown of the increase of plastic deformation after the first stage [39].

### 3.6. Micromorphological Analysis

**Figure 14:**
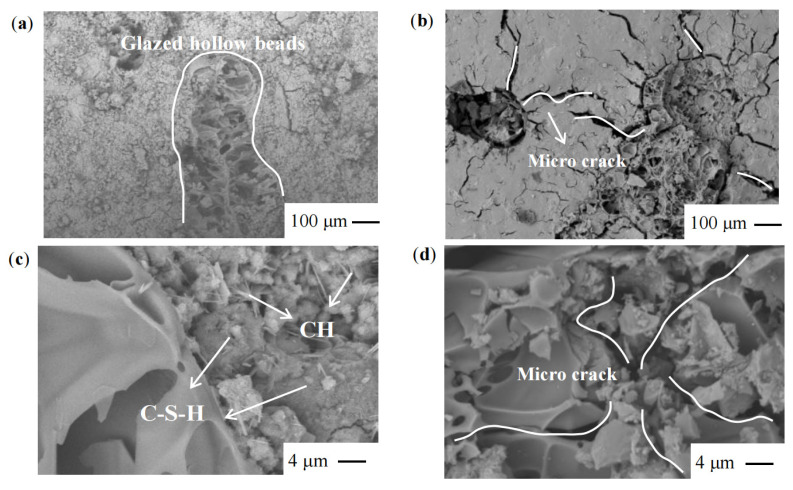
Microscopic morphology of (**a**,**b**) uncycled loaded, (**c**,**d**) cyclic loaded damage grouted material.

For the purpose of microscopic morphological examination, specimens that had not been subjected to cycled loading and those that had suffered damage as a result of cyclic loading were both sampled. See Figure 14.

As can be seen in Figure 14c, in the absence of cyclic loading, the hydrated calcium silicate cementitious material (C-S-H) generated by both cement hydration and calcium hydroxide (CH) reaction with sodium silicate is tightly connected with the CH produced by cement hydration in the shape of plate-like crystals [40]. On the other hand, the overall bonding degree of the products generated by the reaction of glazed hollow beads with cement and sodium silicate is better. It can be seen from Figure 14d that after the cyclic loading damage, when the glazed hollow beads are not yet destroyed, the cement and sodium silicate products are already damaged, cracks are produced, and the cracks are dispersed outward with the glazed hollow beads as the centre, which means that the damage starts from the intersection of the glazed hollow beads and the cement and sodium silicate products, which is because the glazed hollow beads themselves have low strength and are more prone to breaking than the cement and sodium [41]. Stress concentration occurs. This is due to the low strength of the glazed hollow beads, which prevents them from supporting the force that is transmitted from the cement and sodium silicate materials. The occurrence of stress concentration, which, in turn, results in the deterioration of the contact surface first, demonstrates that the function of glazed hollow beads in the bearing is quite restricted.

## 4. Conclusions

The new grouting material has a compressive strength of up to 20.3 MPa after curing for 90 days, and each group of specimens has a higher firmness rate than 0.94. The new thermal insulation grouting material performed optimally when the water-cement ratio was 0.6, the content of glazed hollow beads was 55 kg/m^3^, and the volume ratio of sodium silicate and cement slurry was 0.4. Engineering design can increase strength by increasing the water-cement ratio and reduce the setting time by adding sodium silicate.

Grouting material in the loading-unloading process causes fatigue failure, resulting in cyclic loading and unloading strength being lower than uniaxial compressive strength. Deformation due to fatigue The hysteresis area and width of a single cycle increase in significance as the load stress level increases. For multiple cycles, excluding the failure stage, the curve appears sparse at first and then dense at the end of each cycle load, and the deformation of the specimen occurs primarily in the first cycle, which is represented by a large opening on the curve, and then the density of the hysteresis curve gradually decreases and tends to be stable. When the cyclic loading stress is close to failure, the fatigue compressive strength rapidly decreases after a certain number of cycles, and fatigue failure occurs in the specimen. It is advisable to avoid multiple loadings of the same load at the construction site.

The loading strain is more sensitive to stress changes than the residual strain, and the relationship between the residual strain and the stress is more linear, whereas the loading stress shows a stepped increase, indicating that when the stress changes abruptly, the residual strain accounts for a small portion of the loading strain and the elastic strain accounts for the majority of it.

The fitting correlation coefficients for total output energy and elastic energy were both above 0.71, but the ratio of dissipative energy to energy consumption was low, indicating that the total input energy density and elastic energy density increased as a power function with cyclic stress. Total input energy density increases the fastest with increasing stress level, followed by elastic energy density, dissipated energy density, and energy dissipation ratio under the same stress load. The value of each cycle fluctuates significantly in the early stages but tends to stabilize in the middle and late stages. The dissipative energy density and energy dissipation ratio decrease first and then increase with increasing stress. The deformativity density increases first and then decreases with increasing stress.

Additionally, the plate crystals of Ca(OH)_2_, Ca(OH)_2_, and C-S-H are tightly connected to strengthen the load-bearing capacity of the material. When cycle loading causes damage, the fractures expand outward with the glazed hollow beads serving as the centre of the damage. This causes the interfacial link to be destroyed, which in turn causes the macroscopic damage to the specimen.

## Figures and Tables

**Table 1:** Technical parameters of P·C 42.5 cement.

Fineness (m^2^·kg^−1^)	Loss on Ignition (%)	Water Requirement of Standard Consistency (%)	Setting Time (min)		Compressive Strength (MPa)		Stability
			Initial	Finial	3 Days	28 Days	
342	3.5	25.9	165	220	29.9	49.75	Conformity

**Table 2:** Physical and mechanical properties index of glazed hollow beads.

Particle Size (mm)	Bulk Density (kg·m^−3^)	Apparent Density (kg·m^−3^)	Cylinder Compression Strength (MPa)	Thermal Conductivity (W/(m·K)^−1^)	Fire Resistance (°C)	1 MPa Pressure Volume Loss Rate (%)
0.5–1.5	80–120	80–130	≥150	0.032–0.045	1280–1360	38–46

**Table 3:** Physical parameters of sodium silicate.

Product Model	Silicon Dioxide (SiO_2_) (%)	Sodium Oxide (Na_2_O) (%)	Concentration (Bé)	Elastic Coefficient
SP38	27.3	8.54	38.5	2.25

**Table 4:** Factor level table.

Factor Level	Water-Cement Ratio	Glazed Hollow Beads Content (kg/m^3^)	Volume Ratio of Sodium Silicate to Cement Slurry
1	0.6	25	1.0
2	0.8	35	0.8
3	1.0	45	0.6
4	1.2	55	0.4

**Table 5:** The compressive strength, gelation time, and stone rate of each group of materials.

Mix	Compressive Strength (MPa)							Gelation Time (s)	Stone Rate
	3 d	7 d	14 d	21 d	28 d	60 d	90 d		
1	2.5	6.1	9.3	10.1	11.3	11.6	12.3	301	0.960
2	2.6	7.0	9.5	11.9	16.1	17.2	17.4	313	0.960
3	3.7	9.0	13.5	14.5	19.9	20.2	20.2	233	0.980
4	13.6	14.2	15.1	16.1	16.3	16.6	16.9	138	0.990
5	2.1	3.1	5.3	8.1	9.1	9.9	12.3	209	0.970
6	1.9	2.7	2.9	3.8	3.8	5.4	6.8	413	0.941
7	2.3	13.3	13.6	14.9	15.7	17.3	19.9	100	0.960
8	1.8	3.1	5.1	5.6	5.8	11.4	12.3	177	0.960
9	1.8	2.9	3.9	7.0	9.3	9.8	10.1	99	0.951
10	4.6	8.0	8.5	9.1	9.4	10.7	11.3	72	0.903
11	0.8	1.3	1.5	1.6	1.7	2.5	2.5	456	0.941
12	1.0	1.8	1.8	2.2	2.5	3.3	3.1	310	0.960
13	3.3	7.5	7.6	7.8	8.1	8.4	9.0	77	0.960
14	1.2	1.5	1.5	2.1	2.5	6.2	6.3	213	0.960
15	0.8	1.0	1.4	1.5	1.5	1.7	1.9	361	0.960
16	0.3	0.6	0.6	0.7	0.7	0.8	0.8	1277	0.960

**Table 6:** Fitting formula of energy density parameters and stress level of specimens with different loading cycles.

Number of Loads	Total Input Energy *U*-Stress	Elastic Energy *U_E_*-Stress
1	$y=0.0566x^{2}-0.1444x+0.2606$	$y=-0.0156x^{2}+0.2189x-0.2575$
	$R^{2}=0.9916$	$R^{2}=0.9819$
2	$y=0.0274x^{2}-0.0643x+0.2323$	$y=0.0140x^{2}+0.0277x-0.0171$
	$R^{2}=0.7297$	$R^{2}=0.9965$
4	$y=0.0578x^{2}-0.2260x+0.4377$	$y=0.010x^{2}+0.0694x-0.0757$
	$R^{2}=0.7324$	$R^{2}=0.9968$
6	$y=0.0533x^{2}-0.1924x+0.3742$	$y=0.0108x^{2}+0.0599x-0.0499$
	$R^{2}=0.7166$	$R^{2}=0.9978$
Number of loads	Dissipative energy *U_D_*-stress	Energy consumption ratio *η*-stress
1	$y=0.0723x^{2}-0.3632x+0.5181$	$y=0.1433x^{2}-0.8405x+1.4648$
	$R^{2}=0.9844$	$R^{2}=0.7933$
2	$y=0.0134x^{2}-0.0920x+0.2494$	$y=0.0432x^{2}-0.3857x+1.0799$
	$R^{2}=-0.1953$	$R^{2}=0.2060$
4	$y=0.0478x^{2}-0.2954x+0.5134$	$y=0.1059x^{2}-0.7355x+1.4618$
	$R^{2}=-0.0100$	$R^{2}=0.2484$
6	$y=0.0425x^{2}-0.2524x+0.4242$	$y=0.0880x^{2}-0.5895x+1.1595$
	$R^{2}=-0.0076$	$R^{2}=0.1369$

**Table 7:** Differences in energy competition coefficients at each stage of different loading cycles.

Number of Cycles		Stage 1	Stage 2	Stage 3	Stage 4	Stage 5	Stage 6
1	Total difference	0.288					
	Stage difference						
2	Total difference	1.727					
	Stage difference	1.592	0.258	0.706	0.13	0.035	0.079
4	Total difference	2.269					
	Stage difference	2.182	0.228	0.115	0.097	0.084	
6	Total difference	2.248					
	Stage difference	2.211	0.143	0.11	0.084	0.055	

## Data Availability

Data will be made available on request.
